# Tumours of the Breast

**Published:** 1904-07-16

**Authors:** 


### TUMOURS OF THE BREAST.

MR. ERNEST SHAW,[1] in a paper read before the Abernethian Society, gives a classification of breast tumours in which he combines the practical division, into innocent and malignant, with the more exact method based on histological characteristics. He deals seriatim with the various tissues entering into the formation of the mammary gland, and describes the different growths which may originate in them. The epithelial cells may grow (*a*) outwards or (*b*) inwards. In the former case the new growth may imitate closely the normal gland characters and a fibroadenoma result, or disorderly growth of cells may break through the basement membrane and invade adjacent structures, forming a carcinoma.

In case (*b*) an orderly growth is a duct papilloma; a disorderly invasion is a duct carcinoma.

Fibroma and sarcoma grow from the fibrous tissue of the breast; lipomata, angiomata, and perhaps neuromata may occur in connection with the fatty, vascular, and nervous elements. Secondary carcinomatous and sarcomatous nodules are sometimes found in the breast. Combinations of separate tumour formations are occasionally met with. One breast contained a sarcoma, a fibroadenoma and a nearly pure fibroma.

Mr. Shaw draws careful distinctions between the different forms of cysts with intracystic growths which, in the breast, cause so much confusion because of inaccurate descriptions and uncertain nomenclature. A cyst formed from a dilated duct may contain an adenomatous mass, the gland tubes of which have dilated; the intervening fibrous tissue broken down, and the broken ends projecting freely into the enlarged glandular spaces thus formed, resemble the processes of the next form, duct papilloma.

Duct papilloma is an epithelial ingrowth into the lumen of a duct. Cystic tumours occur near the nipple, they contain a little bloodstained fluid and a soft reddish mass attached to one wall and composed of delicate processes of loose fibrous tissue, and blood-vessels, the whole covered by a layer of columnar cells. Bleeding from the nipple and a tendency to malignant degeneration characterise these two growths. Duct carcinoma consists of a distinct papillary ingrowth within small cysts, and an infiltrating columnar-celled downgrowth; perhaps a malignant stage of duct papilloma.

Intracystic adenofibroma consists of gland tissue in the form of cystic spaces and masses of connective tissue polypoid in form, and either cellular and soft or fibrous and hard projecting into these cysts. This growth may rapidly progress and intracystic adeno-sarcoma result. In one part of such a tumour there will be cysts with fibrous or myxomatous intracystic growths, in another part the cysts have disappeared, and a solid softish mass mottled with extravasated blood is found.

Occasionally there is a smooth thick-walled cyst into the cavity of which projects a small firm irregular nodule of carcinoma. It is not rare to find carcinoma beginning in the outer part of the wall of an apparently simple mammary cyst. The correct surgical treatment of cysts occurring in the breast, judging by pathological experience, is excision rather than incision and drainage.

[1] St. Bart.'s Hosp. Jour., June 1904.

